# Supplementary material for: Metagenomic binning with assembly graph embeddings
Source: Bioinformatics. 2022 Aug 16;38(19):4481–7. doi: 10.1093/bioinformatics/btac557 (PMC9525014; doi:10.1093/bioinformatics/btac557)
Supplement: btac557_supplementary_data [file btac557_supplementary_data.zip › amber outputs.zip!/amber outputs/strong100/genome/vamb/purity_vs_bin_size_SAMPLEID.pdf]

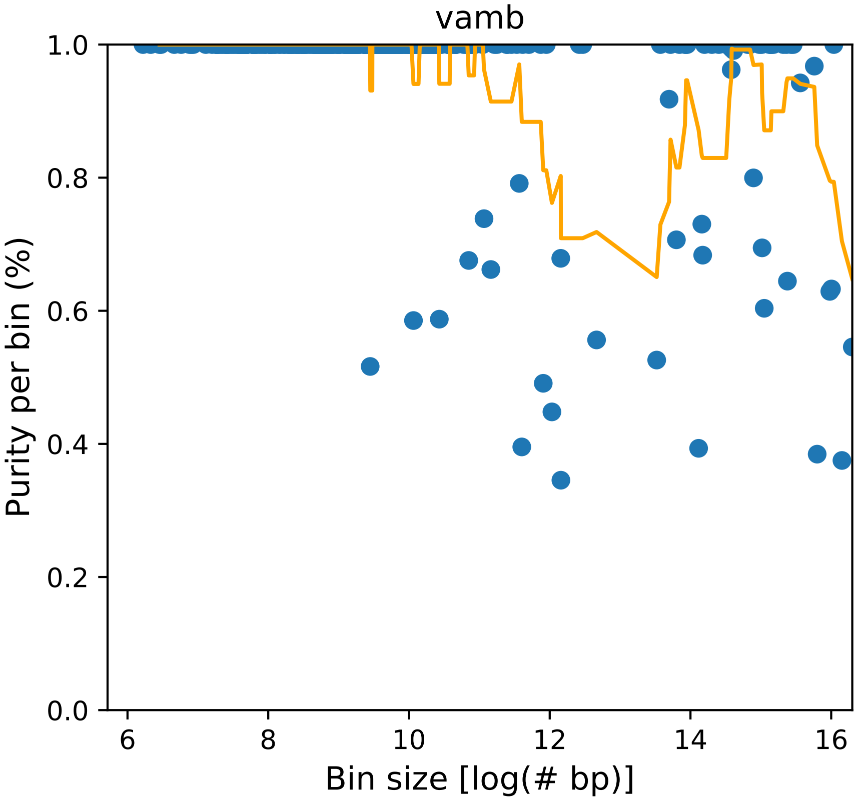

vamb
Purity per bin (%)
Bin size [log(# bp)]
1.0
0.8
0.6
0.4
0.2
0.0
6
8
10
12
14
16
